# Compliance with World Health Organization COVID-19 preventive behaviors in rural counties in Western Kenya: a cross-sectional study

**DOI:** 10.11604/pamj.2024.47.30.40558

**Published:** 2024-01-24

**Authors:** Caleb Nyaranga, Cholo Wilberforce, Fletcher Njororai

**Affiliations:** 1 Department of Public Health, School of Health Sciences, South Eastern Kenya University, Kitui, Kenya; 2 Department of Public Health, School of Public Health and Biomedical Sciences, Masinde Muliro University of Science and Technology, Kakamega, Kenya; 3 Department of Public Health, School of Health Professions, The University of Texas at Tyler, University Boulevard, Tyler, United States of America

**Keywords:** Compliance, COVID-19, mitigation measures, latent class, Kenya

## Abstract

**Introduction:**

the World Health Organization (WHO) recommended various measures to tackle COVID-19, and were adopted by many governments, targeting behavior change among citizens to lower the transmission. There was a paucity of data on the patterns of compliance with different measures within individuals and whether people adhere to all recommended measures or cautiously prefer few but not others. Understanding compliance behaviors and associated factors is important for developing interventions to increase compliance.

**Methods:**

cross-sectional study was conducted among adults in the western region of Kenya. A sample of 806 participants was selected using a stratified sampling method. A structured questionnaire was used to gather data from the participants. Compliance was assessed with six behaviors: hand sanitation, proper hygiene, no handshaking, social distancing, and other guidelines. Latent analysis was used to identify behavioral patterns. Descriptive statistics were used to assess demographic characteristics, in terms of frequency distribution, and percentages. Multinomial logistic regression was used to assess the association between demographic characteristics and compliance level.

**Results:**

compliance was highest for masking (85.3%), and was lowest for social distancing (60.2%). The majority of participants were found to be full compliers (class 1: 40.5%), there was an increased probability of full compliance among those aged between 18-30 years (OR= 1.042; 95% CI: 0.307-13.052, p < 0.040) compared to those aged ≥70

**Conclusion:**

using facemasks had the highest rate of compliance, followed by hand sanitization and proper hygiene. However, overall, the findings showed that while compliance with some protocol behaviors is high, individuals comply consistently across recommended compliance behaviors.

## Introduction

The COVID-19 pandemic spread rapidly across the globe, and by February 2020 it had reached many sub-Saharan African countries [1]. Sub-Saharan African countries such as Kenya have faced a huge challenge in COVID-19 epidemic management and containment due to high levels of poverty and poor health systems [2]. The success of measures to slow or stop the spread of COVID-19, such as wearing face masks and social distancing, depends on the commitment and capacity of individuals to comply with them and change their behavior accordingly [1-3]. Ineffective compliance with these measures can put the achievement of policy outcomes at risk [3]. Unpreparedness for a global plague of immense impact, the COVID-19 pandemic, proved very costly for the world [4]. Suddenly, the world was connected by the desire to find quick solutions all around by pulling efforts, rapid sharing of information, mobilizing resources, and health agency's recommendations of various measures to help slow the rapid spread of the virus [5-8]. Many public health measures have been employed by different countries globally with differing degrees of success in controlling the spread of the COVID-19 pandemic, especially in the absence of vaccines, therapeutics, and treatment [9]. In the early phases of the outbreak of COVID-19 pandemic, governments around the world endeavored to control it through a plethora of containment measures such as hand hygiene and disinfection, isolation of sick people, tracing and quarantine of contacts, and unprecedented mobility restrictions [10].

The World Health Organization (WHO) recommended preventive measures that include social distancing, wearing masks, and regular handwashing with soap or hand sanitization using approved alcohol-based sanitizers. Although these measures are deemed effective at reducing transmission of the disease, they largely depend on voluntary action by members of the public [11]. Understanding compliance behaviors and associated factors is an essential step to have effective and potentially tailored communication plans or measures that are context-specific, and other tailored strategies in dealing with a deadly pandemic or infectious disease [12,13]. World Health Organization COVID-19 public health measures require behavioral changes on the part of the individuals, and communities with varying degrees of compliance influenced by many factors [9]. As such, research on compliance in different populations, communities, and contexts with COVID-19 preventive measures is necessary in identifying effective strategies, barriers, and lessons for continued surveillance and preparedness of current and future pandemics [14]. Interest in this line of work has been ongoing since the onset of the pandemic, and several studies have been published on determinants and predictors of compliance [15-17]. These studies have reported various levels of compliance, ranging from a comparatively high (52%) level of compliance among adults in the United Kingdom [18] to very poor (16%) compliance in parts of Africa [19]. Studies on compliance with WHO public health measures in rural communities in Africa are limited, this study contributes to the base of knowledge for compliance towards COVID-19 prevention and control. Previous studies on public health measures, compliance are more in high-income countries and fewer in Africa [5]. Though some literature exists for low-income countries, there still exists a paucity of literature on compliance with WHO COVID-19 preventive protocols. This study sought to investigate compliance with COVID-19 preventive protocols and associated demographic and economic factors in selected rural counties in western Kenya. Our findings may be helpful for pandemic preparedness through understanding context specific effectiveness of public health measures, support policy decision-making as governments prepare for future epidemics or future waves of the pandemic; and as an addition to the growing evidence that public health measures are a powerful public health tool.

## Methods

**Research design:** this study employed a cross-sectional design in which quantitative research strategy was used to collect data on compliance with WHO COVID-19 behavior pattern and associated demographic and economic factors in the three selected counties in Western part of Kenya. The study was conducted in May-August 2021, at the peak of COVID-19 globally.

**Study setting:** this study was conducted in Kakamega, Vihiga, and Kisumu Counties in western Kenya. Kakamega County covers an area of approximately, 3,050.3 km^2^. The county has twelve sub-counties, eighty-three locations, two hundred and fifty sub-locations, one hundred eighty-seven Village Units and four hundred Community Administrative Areas. There are 433,207 households with an average size of 4.3 persons per household and a population of 1,867,579. Vihiga County lies in the Lake Victoria Basin and covers an area of 531.0 km^2^. Vihiga County is located around 80 km northwest of Eldoret, around 60 km north of Kisumu, and approximately 350 km west of Nairobi City, the capital city of Kenya. It has a population of 590,013 of which 51.9% are females while male constitutes 48.1%. Sixty-four-point four percent (64.4%) of the total population are under the age of 30 [19]. The County has five administrative sub-Counties. The county is further subdivided into 38 locations, and 131 sub-locations. Kisumu County is bordered to the north by Nandi County and to the North East, Kericho County. The land area of Kisumu County totals 2085.9 km^2^ [20,21]. It has a population of 1,155,574 of which Women are made up 50.1% of Kisumu´s population and men represented 49.9%. Sixty-four percent of the total population are under the age of 25. The land area of Kisumu County totals 2085.9 km^2^ (administratively, the county is divided into 7 sub-counties, and these are further divided into 35 wards [21].

**Study population and participants:** all community members aged 18 and above were eligible for participation in the study.

**Sample size determination:** the sample size was determined using G- power as function of the effect size of 0.1, level of significance of 5% and study power of 80% resulting in a sample size of 806. This method has been used in previous studies [22].

**Sampling procedure:** purposive sampling was used to select the three Counties because they were top 15 leading highest COVID-19 prevalence in Kenya and among the top three leading in COVID-19 prevalence in Western part of Kenya [19]. Two sub-counties were selected from each County using proportionate stratified sampling based on whether urban or rural. Proportionate stratified sampling was applied to select the study subjects from the six sub-counties. Two wards per sub-county were selected using simple random sampling per every sub county. A household list was generated based on administrative location headed by the Chief. Systematic random sampling was then used to select households in the selected wards. A representative of the eligible study subjects or house heads in the selected households were randomly picked to participate in the study (Table 1).

**Table 1:** sampling frame

County	Sampled sub-county	Sub-county population	Wards (population)	Sample	Total sampled individuals
Kakamega	Kakamega Central	188,212	Butsotso central (25744)	149	219
			Mahiakalu (12067)	70	
	Navakholo	**137165**	Bunyala Central (38407)	142	187
			Ingotse-Matiha (12091)	45	
Kisumu	Kisumu Central	174,145	Kondele (48004)	93	130
			Milimani (18902)	37	
	Nyando	161,508	Ahero(31440)	60	121
			Onjiko(30937)	51	
Vihiga	Emuhaya	69,250	North East Bunyore (35908)	36	64
			Central Bunyore (27316)	28	
	Hamisi	148 259	Banja (22535)	45	85
			Tambua (18689)	40	
**Total**					806

**Data sources, instrument, and collection:** a structured questionnaire was used to collect data. The questions were adopted and modified from similar studies [23-28]. In addition, the WHO´s and Kenyan Government Ministry of Health guidelines on COVID-19 infection prevention and control (IPC) were reviewed and used to design the questionnaire [29,30]. The questions were translated in Dholuo and Kiswahili which was back translated to check for consistency. Moreover, Cronbach´s alpha value was calculated to check the tools´ reliability and the value of an item score was 0.89. All prospective respondents were approached, and the nature of the study was explained to them. Those who consented to participate were interviewed by the trained research assistants. Before implementing the field survey, a pre-test study was carried out among 80 adults in two sub-counties that were not part of the sample. This provided information on the internal consistency and coherence of the data collection tool. A few adjustments on questions were made after the pre-test study. The tool was self-administered but those who were unable to read and write were assisted by the research assistants. The study was conducted from May to July 2021. Research assistants were recruited and trained for 2 days on design and conduct of the study, on the content of the questionnaire and ethical issues. The purpose of the training was to harmonize concepts of the study design and content of the tools before being used in data collection. The research assistants were graduates of Public health and were familiar with the area of the study, this was to enable them to access all the sampled wards and households without difficulties. There were lead field researchers who were responsible for supervision of the completeness and consistency of the data collected.

**Variable and measurements:** the variables considered were socio-demographic characteristics such age, gender, level of education, county of residence, area of residence, occupation, income level and compliance behaviors of respondents. The study focused on six compliance behaviors: handwashing, face mask wearing, sanitization, social distancing, cough hygiene/ not touching face, and compliance with other WHO guidelines (lock down and travel policies). Participants were asked for their compliance with these behaviors and their responses were categorized as: not at all, rarely, frequently and always.

**Data analysis:** quantitative data were cleaned using data editing, then coded, and entered into SPSS version 26 programs. Descriptive statistics indicating the frequency, percentage, mean, and standard deviation of participants´ general characteristics, and patterns in WHO COVID-19 prevention guidelines for health behavior were assessed. Compliance variables were treated as ordered categorical variables. Average latent class probabilities and substantive interpretation of the classes were identified. Latent class analysis was performed on six WHO COVID-19 prevention behavior. Latent class analysis has been used to analyze behavioral patterns in previous studies [1,1]. Latent profile analysis accounts for the measurement error, relies on a probability-based approach, and provides a statistical test for the appropriate number of categories [31]. The study did not report Bayesian information criterion (BIC) information and entropy values. Multinomial logistic regression model was used to assess the compliance patterns according to demographic, socioeconomic and characteristics in two-step approach. Multinomial regression is generally intended to be used for outcome variables that have no natural ordering. It is also possible to formulate multinomial logistic regression as a latent variable model, making it more appropriate to relate the compliance pattern and demographic characteristic. All covariates were added to the model simultaneously. The significance level was set at 0.05. Data were presented in tables and a graph.

**Ethical considerations:** ethical approval and permission to conduct the study were sought and obtained from the University of Eastern Africa, Baraton Institutional Research Ethics Committee (IREC) (Approval No. (UEAB/REC/50/03/2021) and the National Commission for Science, Technology and Innovation (NACOSTI) License number (NACOSTI/P/21/10100) in Kenya respectively. Other permission was sought from the County Administrators in all the three Counties to be allowed to access the respondents. Verbal and informed consent was obtained from all the participants before recruitment into the study. All relevant information about the study were communicated to all respondents before the study was carried out. The purpose, nature and research techniques involved in the study and its advantages were explained to the subjects/respondents and their caregivers in a language that they understood obtaining their consent. This is important for the respondents to give consent without coercion, pressure, or undue enticement. The participants were assured that there were no risks by participating in the study; they were not exposed to adverse outcomes or harm to the participants. They were also informed that their participation to participate was purely voluntary and that they could opt out if they so wish to at any time. They were assured of confidentiality, no identifying information was collected or analyzed.

## Results

In this analysis, the focus was on individuals with complete observed compliance behaviors, (n=806; 100%) of individuals participated in the study. No participants had missing information on key demographic data and compliance behaviors that were used to identify latent classes. A total of 806 individuals from three Counties in Western Kenya participated in the study. Slightly over half (52.6%) of the participants resided in Kakamega County followed by 33.9% who resided in Kisumu County while 13.4% of the participants were from Vihiga County. A slight majority (55%) of the participants were males. The mean age of the participants was 35.9 (SD= 13.07) and about 70% of the participants were in the age range of 18-40 years, while the modal age group was 18-30 years. Most of the participants completed secondary school representing 42.9% and only 8.3% had no formal education. More participants (57%) lived in rural areas, and half (50%) of the participants were not employed (Table 2).

**Table 2:** demographic characteristics of the respondents

Variable	Frequency	Percentage
**Gender**		
Male	442	55
Female	364	45
**Age**		
18-30	305	37.8
31-40	264	32.8
41-50	96	11.9
51-60	76	9.4
61-70	45	5.5
>70	20	2.5
**Education level**		
No education	67	8.0
Primary	106	13.2
Secondary	335	42.9
University	298	36.9
**County of residence**		
Kakamega	406	50.4
Kisumu	251	31.1
Vihiga	149	18.5
**Area of residence**		
Rural	461	57.3
Urban	345	42.8
**Occupation**		
Civil servant	113	14
Private sector	96	11.9
Self employed	190	23.6
Non-employed	407	50.5

**Figure 1:**
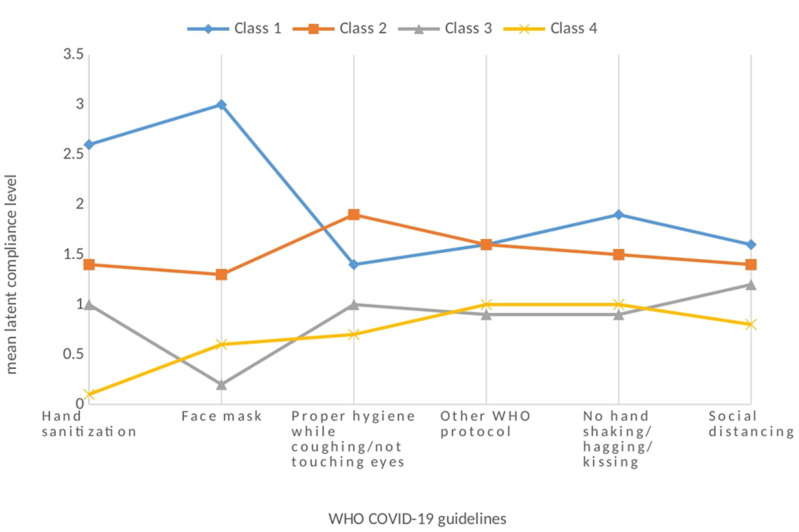
distribution of compliance by World Health Organization protocol/behavior

**Compliance with COVID-19 guidelines:** the distributions of the individual compliance behaviors are displayed in Figure 1. The majority of participants reported frequent or complete compliance for each of the WHO COVID-19 guideline behaviors. Compliance was highest for masking (85.3%), handwashing and sanitization (78.9%), and proper hygiene while coughing to avoid face touching (66.0%). Compliance to other WHO COVID-19 guidelines consisting of travel policies and lock down policies accounted for 62.7%. Compliance was lowest for social distancing (60.2%). The study revealed that (40.1%) of the respondents always fully complied with the WHO guidelines selected for the study.

**Figure 2:**
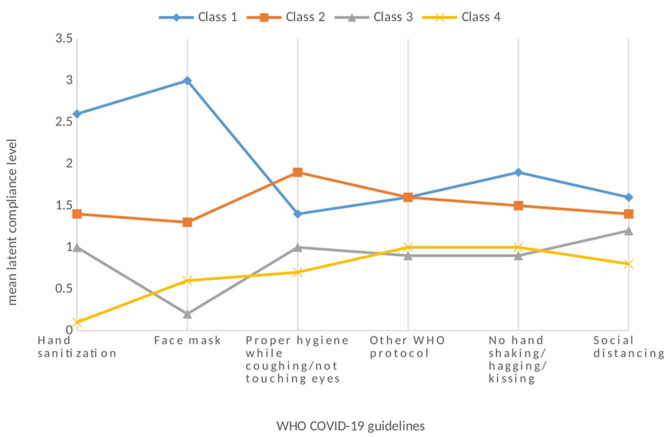
mean latent class by compliance level by World Health Organization protocol/behavior

**Distribution of latent class and compliance level:** as shown in Table 3 below, the majority of participants were found to be full compliers (class 1: 40.5%), reporting high compliance levels with each of the COVID-19 requisite behaviors except proper hygiene. Nearly a third of the participants (class 2: 29.3%) were frequent compliers, exhibiting frequent compliance in all with each of the WHO COVID-19 requisite behavior except proper hygiene while coughing/sneezing/ not touching eyes/nose where these participants exhibited full compliance. In comparison with full compliers, these individuals reported similar compliance with “other” WHO guidelines (travel and lockdown policies) and lower compliance with hand sanitizing, face masking, no hand shaking/hugging or kissing, and social distancing. Infrequent compliers (class 3) constituted 17.7% exhibiting overall least compliance level with face masking, Other WHO guidelines (travel and lockdown policies) and not shaking hands/hugging/kissing. The non-compliers (class 4), the last class represented 12.5% of the participants, had the least compliance level particularly with hand sanitation/washing and social distancing. As presented in Figure 2, latent class profile had different characteristics as per study variables. For instance, Class 1 comprised participants with high compliance with all the COVID-19 compliance behavior including hand sanitation, face mask, not shaking hands. Latent class 1 consisted of 326 participants. Latent Class 4 had 101 participants who were low compliers in all compliant behaviors.

**Table 3:** average latent class membership probability (column) by most likely latent class (row), and expected sample sizes by latent class

	Mean class probabilities				(n %)
Class 1	0.12	0.27	0.18	0.43	326 (40.5)
Class 2	0.52	0.26	0.09	0.13	236 (29.3)
Class 3	0.18	0.38	0.38	0.07	143 (17.7)
Class 4	0.05	0.16	0.69	0.14	101 (12.5)

**Demographic characteristics and compliance patterns from the two-step multinomial regression:** an attempt was made to assess whether those who fully complied with COVID-19 guidelines differed on some characteristics from those who did not fully comply. Those who did not fully comply were reclassified as moderate compliers and constituted classes 2-4; while those who fully complied were class 1. A two-step multinomial regression model was conducted to obtain Odds ratio at 95% Cl. Compared with full compliers, there was statistical evidence that moderate compliers, are younger, less educated, residents of Kisumu and those with lower income. Demographic characteristics and compliance patterns from the two-step multinomial regression, is presented in Table 4. Participants aged 18- 30 years and 31-40 years were each two times more likely to have moderate compliance toward COVID-19 preventive measures than those >70 years old. Comparatively, older age groups; 51-60 years (OR = 2.51; 95% CI, 0.84-3.92, p < 0. 015), and 61-70 years (OR = 2.13; 95% CI, 1. 09-4.23, p <0.021), were more likely to be associated with full compliance than participants who were aged above 70 years, as shown in Table 5.

**Table 4:** demographic characteristics and compliance patterns from the two-step multinomial regression

Factor		Moderate compliance		Full compliance	P-value
		OR(95% CI)	P-value	OR (95% CI)	
**Gender**	Male	1.04(0.65,1.67)	0.87	0.94(0.55,1.63)	0.83
	Female	Reference			
**Age**	18-30	2.00(0.31,13.05)	0.04	0.65(0.09,4.62)	0.66
	31-40	2.041(0.16,6.67)	0.005	0.37(0.05,2.57)	0.31
	41-50	1.89(0.27,13.26)	0.52	0.68(0.09,5.36)	0.71
	51-60	1.92(0.26,14.04)	0.52	2.50 (0.84,3.92)	0.015
	61-70	2.71(0.32,22.97)	0.36	2.13(1.29,4.23)	0.021
	Above 70	Reference			
**Education**	None	1.2(0.94, 5 .52)	0.001	0.43(0.16,1.18)	0.10
	Primary	1.3(1.14, 6.248)	0.002	0.28(0.10,0.74)	0.01
	Secondary	0.72(0.37,1.39)	0.32	0.93(0.45,1.94)	0.001
	Tertiary	Reference			
**County of residence**	Kakamega	0.44(0.17,1.15)	0.10	1.54(0.59,2.55)	0.001
	Kisumu	0.23(0.09,0.59)	0.002	0.18(0.07,0.51)	0.23
	Vihiga	Reference			
**Occupation**	Civil servant	1.37(0.44,4.27)	0.59	1.75(0.49,6.28)	0.39
	Private sector	4.02(2.98-6.50)	0.050	5.76(1.25,26.45)	0.025
	Self-employed	1.05(0.44,2.46)	0.92	1.27(0.47,3.44)	0.64
	Unemployed	Reference			
**Income level**	1000-9999	1.36(0.50,3.72)	0.54	0.92(0.29,2.92)	0.89
	10000-19999	2.32(0.66,8.18)	0.19	1.71(0.42,6.95)	0.45
	20000-29999	0.71(0.27,1.85)	0.49	0.62(0.20,1.88)	0.395
	30000-39999	1.09(0.34,3.54)	0.89	0.41(0.10,1.78)	0.23
	40000-49999	0.00 0.00	0.000	0.00 0.00	0.00
	50000-59999	0.51(0.12,2.11)	0.35	0.58(0.12,2.83)	0.50
	≥60000	0.64(0.15,2.74)	0.55	1.73 (0.15,3.64)	0.002
	No income	Reference			

## Discussion

This study investigated compliance with WHO COVID-19 preventive protocols in communities residing in four counties in western Kenya. Compliance was assessed across six constructs including use of facemask, hand sanitization, cough hygiene and avoiding physical contacts. These protective behaviors were related to concern over the severity of infection. Studies elsewhere showed that people tended to decrease their daily contacts, reduce trips to crowded places, and wear masks when leaving home during COVID-19 outbreaks [32]. Overall full compliance in this study was 40%. Full compliance in our study was lower than 52.5% reported among UK adults [19]. This difference could be accounted for by geopolitical and social differences in development between the two countries, given that the United Kingdom is a high-income country. This study found that using facemasks had the highest rate of compliance (85.3%), followed by self-reported for hand sanitization/ washing (78.9%), and proper hygiene while coughing/ avoid face touching (66.0%). There was a stark contrast between these findings when compared with a study done in Ethiopia which reported facemask use rate to be lowest at 56%, hand hygiene to be highest at 97% while cough hygiene was at a high of 91% [33]. This shows that there was a relatively higher level of compliance with most behaviors and lower compliance with social distancing. Individuals comply consistently across recommended compliance behaviors. Consistent with previous studies [19,3,4] this study suggests that people are motivated differently to comply with the WHO protocol or compliance behavior in different settings. The lower compliance to social distancing could be attributed to its opportunistic nature, for example when meeting relatives and friends and the desire to avoid isolation and loneliness which is a common human nature to be social. This is especially so for communal collective societies such as most African societies/cultures, where people tend to be together more. Other studies have noted that people may integrate physical distancing into their lifestyle or postpone some activities until an epidemic wane [35]. Simulations in other studies have suggested that if people are in a state of panic such as panic-buying in a crowded area, physical distancing is of little use [32]. Using social incentives like material incentives, knowledge sharing activities and support to educate people on the importance of physical distancing would be suitable in this kind of situation to inhibit spread of the virus. This demonstrates the diverse nature of behavioral compliance and warrants context-specific studies and interventions.

Furthermore, compliance with face mask use in this study was lower than that reported by a study earlier conducted in Kenya, which reported a 97% compliance rate [36]. The difference here could be accounted for by the demographic differences between the two studies. While the referenced study was among youths with nearly 80% of the sample aged between 18-29 years, in our study this demographic only represented 37% of the sample. Our analysis did indicate that there were increased odds of moderate to frequent compliance among this age bracket. This study determined four groups of compliance behavior using latent class analysis: the full, frequent/moderate, infrequent, and non-compliers. It also provides a flexible approach to determine the number of potential categorical variables to model observed variables within the categories. Latent profile analysis accounts for the measurement error, relies on a probability-based approach, and provides a statistical test for the appropriate number of categories [3,1]. Consistent with a study in London [11,3,7] generally some behaviors, such as face mask wearing, and hand-washing and sanitization were performed more frequently by most individuals, however, there were almost consistent levels of compliance across all the compliance behaviors.

A small minority comprising 12.5% of individuals were non compliers and particularly reported low compliance levels on face mask wearing, handwashing and sanitization and other WHO COVID-19 guidelines which composed of (travel and lockdown policies) and not shaking hands/hugging/kissing. The study revealed that compliance with individual behaviors were each related to individual characteristics. There was evidence that full compliance was strongly related to older age, participants working in private sector, higher income, and higher level of education. Younger adults had lower levels of compliance than older adults. Age as predictor of compliance behavior has been noted in other studies [19,37,38]. This could be due to lower risk perception, attributed to lower perceived susceptibility, among the young adults, especially males [39]. Younger male tend to go out and get together with their peers without using a mask and physical distancing compared to female [24]. Other studies have reported that many younger people disregard most COVID-19 prevention guidelines/ rules because they think the disease is less dangerous for them [39]. On the contrary, currently, older people are classified as being at moderate risk from COVID-19 irrespective of their general health and this could increase their risk perception and most of the time challenged by requirements to spend more time at home. This behavior would increase the transmission of COVID-19 infections. The finding that other demographic characteristics including level of education, income level and private sector employee strongly related to high compliance is also not consistent with previous studies in the literature, which generally show that level of education, high income level, and gender do not explain difference in compliance levels [21,37,39].

**Strength and limitations:** the present findings were based on a large sample which is a more representative of population from different communities and Counties and thus, more generalizable. The study used a priori analysis to determine the sample size allowing the investigators to determine the study power before the study commenced. The study had statistical power to detect sufficient effect size. Furthermore, while the COVID-19 associated measures in Kenya are mostly in line with WHO recommendations, there are important differences in policies and information which may influence noncompliance. Non-compliance with certain measures may therefore vary by the specific public health campaigns and official recommendations in each region. However, there are some limitations to our study that need to be noted. One limitation is our sample was mainly from western Kenya, relatively young, and slightly more males. The study used cross-sectional data, so results may be biased by unobserved confounding variables as the pandemic evolved and the statistical associations generated are not causal. Another limitation of the present work is that compliance behaviors were self-reported voluntarily by participants. The study relied on participants to reflect on and report the extent to which they engage in these behaviors. According to self-reported measures differ in their similarity with the true behaviors, as participants may be unwilling to report accurate behavior.

## Conclusion

This study found that using facemasks had the highest rate of compliance, followed by hand sanitization/washing and proper hygiene while coughing/avoiding face touching. However, the overall findings showed that while compliance with some protocol behaviors is higher in general, individuals comply consistently across recommended compliance behaviors which is congruent with previous studies. Demographic factors (age, gender, education, and income) are important factors that influence compliance behavior on infectious diseases. Overall, our findings suggest that old people were largely complying with COVID-19 public health measures during the initial wave of the pandemic. This knowledge can be used in planning interventions for infectious disease outbreaks like COVID-19.

### What is known about this topic

- *Coronavirus disease 2019 is a respiratory disease caused by the highly contagious novel coronavirus which overwhelmed the world;*
- *Governments implemented various measures to address COVID-19, focusing on changing citizens’ behaviours in order to lower the transmission of the virus;*
- *Compliance with infection prevention and control (IPC) protocols is critical in minimizing the risk of coronavirus disease (COVID-19) infection among individuals*.

### What this study adds

- *Individuals display consistent levels of compliance across various World Health Organization public health measures, however, compliance was lowest for social distancing;*
- *Overall, our findings suggest that old people were largely complying with COVID-19 public health measures during the initial wave of the pandemic; this knowledge can be used in planning interventions for infectious disease outbreaks like COVID-19*.
